# Bacterial, Gut Microbiome-Modifying Therapies to Defend against Multidrug Resistant Organisms

**DOI:** 10.3390/microorganisms8020166

**Published:** 2020-01-24

**Authors:** Amy Feehan, Julia Garcia-Diaz

**Affiliations:** 1 Infectious Disease Department, Ochsner Clinic Foundation, New Orleans, LA 70121, USA; amy.feehan@ochsner.org; 2 The University of Queensland Faculty of Medicine, Ochsner Clinical School, New Orleans, LA 70121, USA

**Keywords:** fecal microbiota transplant, multidrug resistant organisms, antibiotic resistance, prebiotic, probiotic

## Abstract

Antibiotics have revolutionized human and animal healthcare, but their utility is reduced as bacteria evolve resistance mechanisms over time. Thankfully, there are novel antibiotics in the pipeline to overcome resistance, which are mentioned elsewhere in this special issue, but eventually bacteria are expected to evolve resistance to most new compounds as well. Multidrug resistant organisms (MDROs) that cause infections increase morbidity, mortality, and readmissions as compared with susceptible organisms. Consequently, many research and development pipelines are focused on non-antibiotic strategies, including fecal microbiota transplantation (FMT), probiotics and prebiotics, and a range of therapies in between. Studies reviewed here focus on efforts to directly treat or prevent MDRO infections or colonization. The studies were collected through clinicaltrials.gov, PubMed, and the International Conference on the Harmonisation Good Clinical Practice website (ichgcp.net). While the gold standard of clinical research is randomized controlled trials (RCTs), several pilot studies are included because the field is so young. Although a vast preclinical body of research has led to studies in humans, animal and in vitro studies are not within the scope of this review. This narrative review discusses microbiome-modifying therapies targeting MDROs in the gut and includes current results, ongoing clinical trials, companies with therapies in the pipeline specifically for MDROs, and commentary on clinical implementation and challenges.

## 1. Introduction

The gut serves as a reservoir of multidrug resistant (MDR) pathogens, particularly in patients who have had extensive antibiotics or a long hospital stay [1]. While most patients who become colonized do not progress to a symptomatic infection, certain patient populations are particularly vulnerable to multidrug resistant organisms (MDRO) infections, including immunosuppressed patients [2,3], travelers [4], patients in intensive care units [5], and those in long-term care facilities [6]. Additionally, those who are colonized can transmit MDROs to patients vulnerable to infection in the hospital setting. Antibiotics kill subsets of commensal gut bacteria and allow for the unchecked growth of pathogenic species, causing antibiotic use to be a major risk factor for MDRO colonization and infection [7].

Most of the microbiome-modifying therapies, or non-antibiotic therapies intended to change the bacterial makeup of the gut microbiome, are meant to replace good bacteria and out-compete pathogenic bacteria to regrow a healthy microbiome [8,9,10]. This discussion excludes developments in the bacteriophage and lysin field, although these are also capable of modifying the gut microbiome. Fecal microbiota transplant (FMT) and donor-derived therapies are beginning to become more heavily regulated, while probiotic and prebiotic or food supplement-based strategies remain unregulated. Guidelines have been developed to use FMT safely and effectively [11]. While some probiotics have disease-specific recommendations [12], dosing is still difficult. While a handful of clinical trials demonstrate some efficacy for preventing colonization or transmission of MDROs, much more evidence is needed to support the use of FMT, probiotics, or prebiotics in specific patient populations with or without adjunctive therapies. This review is intended to give clinicians and clinical researchers a context for a number of cutting-edge therapies to reduce MDRO carriage and infection.

## 2. Methods

Clinicaltrials.gov was searched for trials containing the words “fecal”, “probiotic”, and “prebiotic” in the “other terms” field with the “interventional” filter selected. Additionally, the International Conference on Harmonisation Good Clinical Practice website (ichgcp.net) was searched for the same terms. The resulting 2117 studies were manually reviewed for terminology in the title, outcomes, and intervention that included any of the following terms: fecal transplant, fecal microbiota transplant, probiotic, prebiotic, antibiotic resistant, antimicrobial resistant, carbapenem, carbapenem-resistant enterobacteriaceae (CRE), vancomycin, vancomycin-resistant enterococci (VRE), extended spectrum beta-lactamase (ESBL), multidrug resistant organism, MDRO, drug resistant, *Staphylococcus aureus*, and MRSA. Additionally, referenced articles within those studies that directly addressed MDRO prevention, decolonization, or treatment were added to this review. Finally, companies that are developing non-antibiotic therapeutics that target the gut were reviewed and those that had clinical trials for MDRO treatment or prevention are included in this review.

Duplicates were removed, and 82 articles or clinical trial postings were reviewed for eligibility. Twenty-nine of these were removed for irrelevance or a lack of data regarding MDRO outcomes. Fifty-three studies were identified as measuring MDROs or MDRO colonization as the primary outcome. On pubmed.gov, studies were searched for the corresponding clinical trial numbers or study authors to find the results of trials that fit criteria within clinicaltrials.gov. While this is not an exhaustive list of every study with data on MDRO colonization, the studies selected represent interventional trials that address the use of gut microbiome-modifying therapies to alleviate the surge of MDRO colonization and infections.

## 3. Fecal Microbiota Transplantation

The concept of transplanting human fecal material is more than 1700 years old, but modern medicine has only recently begun to seriously consider this therapy [13] with rigorous clinical trials beginning in the 2010s [14]. Despite the overwhelming number of trials without a severe adverse event, this therapy is not without risks. Typically, side effects include abdominal discomfort, fever, nausea, bloating, gas, and diarrhea. However, in June 2019, the US Food and Drug Administration (FDA) released a warning regarding FMT after two immunocompromised patients became seriously ill and one died [15] drawing media attention [16]. There is an ongoing controversy over whether the FDA should heavily regulate FMT, which has traditionally been performed using stool from a friend or family member under the guidance of a physician. As regulations become more rigorous, smaller stool banks will not be able to keep operating while for-profit FMT companies can financially keep up with increased testing and storage restrictions. Increased oversight will ultimately benefit this young scientific field by ensuring that patients get the safest, most effective FMT source material through an effective route of administration.

As of December 2019, 197 studies in clinicaltrials.gov were completed or actively enrolling subjects for FMT therapy, mostly for *Clostridioides difficile* infection (CDI), which was the genesis for treating MDROs with FMT [17]. Twenty-one studies in clinicaltrials.gov are for MDRO decolonization or treatment (Table 1). Companies that are focusing on MDRO-specific indications for their products, or who could use their products for this purpose are listed in Table 2. Several recent reviews have discussed the usefulness of FMT for MDRO decolonization or treatment [18,19,20,21]. The majority are case studies and results are mixed; some studies show complete eradication of MDROs and some show very limited changes in colonization. In a case study of a man who was colonized with 12 MDROs and received FMT for CDI, the result was partial decolonization (four MDROs 15 weeks post-FMT), fewer episodes of sepsis and infections, and less antibiotic use [22]. While this case seemed promising, the only randomized controlled trial (RCT) directly addressing MDRO decolonization found a small, but not significant reduction in ESBL and CRE colonization after antibiotics and FMT [23]. However, the low enrolment number and variability in both colonization and treatment methods (e.g., FMT pill at some sites and nasogastric application at others) could have contributed to the high variability in results. Additionally, there was no consistency in donor stools, with two separate institutions recruiting multiple donors, and the authors note that antimicrobial resistance (AMR) genes could have been acquired following FMT given that the screening process was not exhaustive for all possible contaminants. 

Another small retrospective case-matched study of 20 patients in France showed 80% (8/10) of patients cleared CRE or CRE/*Acinetobacter* colonization 14 days after FMT versus 20% (2/10) in the control group [24]. In other small studies (*n* = 8 to 20) with various endpoints, the effectiveness of decolonization ranged from 20% to 93% [1,25,26,27,28,29]. One study by Davido et al. suggests that based on an 87% decolonization rate of VRE at three months, FMT should be further explored to combat outbreaks within hospitals [30]. However, because only eight people were in the study, larger studies are needed to determine whether FMT could work in the face of a large outbreak of VRE infection.

Oddly, studies of probiotics have more consistently used placebo controls than FMT studies, as discussed in the probiotics section and shown in Table 1 and Table 3. More thorough RCTs are needed to determine the utility of FMT for specific MDROs using single-donor source material.

## 4. Probiotics and Prebiotics

Probiotics are not federally regulated unless companies undergo regulatory compliance checks on their own or want to market their product as a drug. Anyone can sell and market probiotics regardless of whether the bacteria are alive or even present. Most probiotic companies do not genotype their bacteria or assess the presence of AMR genes [32], although Cabana et al. noted that reputable companies follow good manufacturing procedures and test for AMR genes, which can be verified by consumers [33]. While the debate over the safety of probiotic use in healthy individuals is ongoing, several clinicians and researchers have voiced concern over probiotic use in immunocompromised populations [34,35,36,37]. Side effects, even in healthy populations, can include infections, immune stimulation, production of harmful byproducts, and the spread of AMR genes [38]. Because of the lack of regulation for these products, bacteria that are not listed on the label can be present in the product and cause unexpected side effects. Regardless of safety, only a few health conditions have been studied for efficacy rigorously enough to garner a recommendation by expert clinicians [12]. Most prominent in these recommendations for gastrointestinal (GI) disorders, immune response, and liver disease are lactobacillus GG (LGG) and VSL#3, which are also the most extensively studied for MDRO indications thus far. It should be noted that the product, VSL#3, developed by Claudio De Simone at VSL Pharmaceuticals is now sold as VISBIOME® and is materially different from the product that VSL still sells as VSL#3 [39]. Because of this covert change in formulation, several research studies of VSL#3 were terminated, and some countries and retailers pulled VSL#3 from circulation.

Currently, 20 studies have tested or are testing probiotics, and one is testing a prebiotic for MDRO decolonization or prevention (Table 3). Ten of these studies have shown no difference in colonization following probiotic therapy in subjects who were healthy volunteers [40], methicillin-resistant *S. aureus* (MRSA)-colonized [41], VRE-colonized [42,43], ESBL-colonized [31], MDR *Escherichia coli*-colonized [44], a mix of MDRO colonization [45], on antibiotics or a ventilator [46], preterm infants [47], or travelers [48]. While these results can seem dismal, most studies have used LGG or *L. reuteri* singularly, so much more remains to be explored.

One striking outlier is a study done by Manley et al., in 2007, in which pasteurized yogurt with and without LGG was administered [49]. Of the 27 patients enrolled, 23 subjects completed the trial and 11/11 (100%) in the treatment arm were negative for VRE colonization eight weeks after initiation of the treatment as compared with 1/12 (9%) in the control arm. Eight of the control patients were then crossed over to receive LGG treatment, and all of them cleared VRE within four weeks. While this study seemed extremely promising, a larger trial by Warrack et al. that started the following year concluded that there was no difference in VRE colonization rates in recently infected patients who used 6 grams of LGG. Colonization status was only available for a portion of the participants (36/50), and an even smaller number were colonized at the beginning of the study (9/36) [42]. At four weeks, two additional patients in the control arm had become colonized and one in the treatment group had been cleared of VRE. Despite these findings, the same group found some efficacy of a particular LGG strain called HN001. Supplementation with *L. rhamnosus* HN001 reduced MRSA colonization, but these results were mainly restricted to gut decolonization, not nasal or other sites [41,50]. In a study of 61 children colonized with VRE, one group found that LGG temporarily decolonized patients, but ultimately some would become recolonized after treatment stopped [51].

Ongoing trials include a large study at Taipei Medical University that is examining the efficacy of a probiotic cocktail on VRE colonization (NCT03822819), and unlike the Warrick study [42], the researchers directly recruit patients who are colonized with VRE. Another trial examining the efficacy of *Bifidobacterium infantis* in women with recurrent MDRO urinary tract infections is ongoing at Ochsner Medical Center (NCT03644966), but neither of the authors are involved in that work.

In a departure from probiotics, Kaleido Biosciences is assessing KB109 for its ability to clear VRE, ESBL, or CRE colonization (NCT03944369). Their technology consists of a library of nondigestible glycans branded as Microbiome Metabolic Therapies (MMT™) that can be assessed in thousands of patient samples *ex vivo* for a compounds ability to encourage the growth of certain bacteria that outcompete unhealthy bacteria or inflammatory bacteria (reviewed in [52]). Some of the assays that support the use of these compounds for eliminating MDRO carriage were recently presented [53]. Inulin, an oligosaccharide that is undigestible by humans but fermented by bacteria in the gut, is currently being used in an ongoing clinical trial at Columbia University (NCT03865706). Typically, prebiotics cause moderate gastrointestinal issues like bloating and gas, but are unlikely to increased AMR genes because they are not made with live bacteria. Thus, prebiotics could be a valuable therapeutic tool in the fight against MDRO colonization and infection, but their efficacy remains to be determined.

## 5. Clinical Considerations and the Future of Microbiome-Modifying Bacterial Therapies.

Clinical use of FMT has been somewhat regulated; the FDA has exercised enforcement discretion regarding the investigation of new drug requirements for CDI in the face of overwhelming evidence that FMT is highly effective in otherwise treatment-resistant or recurrent CDI. Guidelines published regarding the implementation of FMT for CDI [11] could translate to use for other diseases. However, there is a lack of diagnostic capacity or even a clear understanding of what constitutes a healthy microbiome. It is difficult to determine whether FMT directly causes reliable changes in the microbiome or if specific, rationally designed therapies would work more effectively in such a complex system. Additionally, many studies use related donors, a multitude of donors, or inconsistent screening processes, making it difficult to determine if fecal transplant from any healthy donor is effective or if more specific elements of FMT are necessary for successful engraftment. Recently, super-donors have drawn attention in the field [54], and in the case of irritable bowel syndrome, a super-donor could make the difference as to whether FMT works or not [55]. In this same vein, perhaps therapies could replicate the bacterial consortiums found in super-donor individuals and be used in a standardized manner to compare the therapy to decolonize different organisms. In the clinical world, a diagnostic to help determine when patients are likely to succeed or fail with various FMT products or when they would need additional doses could significantly increase the use and consistency of FMT therapy. In the basic science world, a commercially available assay, such as the platform that Kaleido uses to test the output of various combinations of bacteria, could accelerate new strategies for FMT, prebiotic, and probiotic approaches, and even precision medicine.

Overall, the challenges with clinical implementation of probiotic therapies for MDROs or other diseases stem from a lack of regulation and insufficient scientific studies to determine appropriate dosing or extensively compare strains and strain combinations for MDRO decolonization. Some new studies in animals show that whether the probiotics are living or dead may not even matter [56], and that there may be more efficacy in simply using bacterial byproducts for therapy. Piewngam et al. found that people colonized with *Bacillus* from their diet had zero incidence of colonization with MRSA, which was markedly different from that of the 25% of people without *Bacillus* who were colonized in the GI tract and nasal cavity. Through in vitro testing, they discovered that fengycins, a class of cyclic lipopeptides, are produced by *Bacillus* and directly inhibit the growth of MRSA [57]. While this type of therapy has yet to be developed for a clinical trial, it signifies the value of more rigorous preclinical assessment of probiotics to pinpoint the most efficacious mechanisms of action.

Microbiome-modifying therapies would benefit from robust RCTs that are laser focused on a specific pathogen or patient population and a rational dosing regimen. An equivalent assessment to pharmacokinetic and pharmacodynamic studies, such as daily sequencing of stool samples to assess engraftment, would assist in determining dose and frequency effects, and commercial companies (e.g., CosmosID, CoreBiome, and Viome) have optimized shotgun sequencing of the whole microbiome to be extremely cost effective. Furthermore, if any microbiome therapy is going to be used for MDRO treatment or prevention, clear guidelines for use in gram-positive versus gram-negative infections and various mechanisms of resistance are necessary, as FMT has not been particularly efficacious with MDR gram-negative bacteria so far [44,45]. Some have speculated that FMT could be less effective for ESBL decolonization, although the mechanism by which this is possible has yet to be defined [25,58]. Finally, research needs to show more than a lack of inferiority; rather, studies should be designed to measure outcomes where FMT or probiotic therapies could show an advantage beyond acute treatment (e.g., reduced readmission, fewer subsequent infections, and fewer long-term complications). A 2019 study from Finland has suggested that extensive treatment with antibiotics can induce Parkinson disease 10 to 15 years later [59], and although long-term studies are costly, perhaps the long-term implications are worth considering for lengthy therapy options.

## 6. Conclusions

Given a handful of positive results of bacterial therapy for MDRO colonization, this strategy is certainly worth pursuing in a systematic rigorous way. Studies that include single-source FMT or standard, regulated probiotic therapies for specific MDROs enable physicians and hospital systems to make rational decisions about how to best handle colonized or infected patients and healthcare workers. However, these treatments alone will not solve the issue of antibiotic resistance. Other therapies including bacteriophage [60,61], lytic enzymes [62], novel cleaning techniques [63], repurposed drugs with antibiotic activity [64], and bacterial byproducts [57] should be further developed. With enough duplicity in treatment options, hopefully physicians will be able to prevent global outbreaks of treatment-resistant infections.

## Figures and Tables

**Table 1:** Clinical trials for fecal microbiota transplantation (FMT) that directly target multidrug resistant organisms (MDRO) colonization or infection.

Year	Sponsor	Product	Placebo/Controlled	Clinical Stage	Target	Number of Patients	NCT Number	Effect	Conclusions	Reference
2015	Washington University School of Medicine	FMT (enema)	no	Phase 1	MDRO infections	20	NCT02312986		Ongoing	
2015	Jinling Hospital, China	FMT (nasointestinal tube)	no	N/A	MRSA	10	NCT02390622		Ongoing	
2016	University of Miami	FMT (enema)	no	Phase 1	MDRO	20	NCT02816437		Ongoing	
2016	Cepheid/ Emory University	Allogeneic FMT	yes	Phase 1	MDRO	20	NCT02922816		Ongoing	
2017	Raymond Poincaré Teaching Hospital	FMT (nasoduodenal tube)	no	N/A	CRE/VRE	8	EudraCT 2014-003048-11	++	3/8 patients decolonized after 3 months	[27]
2017	Medical University of Warsaw	intraduodenal FMT	no	N/A	MDRO	20	NCT02461199	+++	15/20 (75%) complete decolonization	[1]
2017	Raymond Poincaré Teaching Hospital	FMT (nasoduodenal tube)	no	N/A	VRE	8	NCT03029078	++++	87.5% eradication at 3 months	[26]
2017	Microbiome Health Research Institute	Autologous FMT	yes	Phase 1	MDRO	4	NCT03061097		Completed. No results available.	
2017	Microbiome Health Research Institute	FMT (pill)	yes	Phase 2	VRE	9	NCT03063437		Completed. No results available.	
2017	Seattle Children’s Hospital	FMT	no	Phase 1	ESC-R Enterobacteriaceae	20	NCT02543866		Ongoing	
2018	Academic Medical Centre, Amsterdam	FMT	no	N/A	ESBL	15	ISRCTN48328635	++	20-40% clearance with one or two FMTs	[29]
2018	Versailles Saint-Quentin University	FMT (nasoduodenal tube)	no	N/A	CRE/VRE	17		+++	4/8 CRE clearance 7/8 VRE clearance at 3 months	[28]
2018	Rebiotix	RBX2660	no	Phase 1/2	Recurrent MDRO UTIs	60	NCT03367910		Ongoing	
2018	Chinese University of Hong Kong	FMT	yes	Phase 2	CRE/VRE	40	NCT03479710		Ongoing	
2018	Rambam Health Care Campus	FMT	no	N/A	CRE	60	NCT03167398		Ongoing	
2019	University Hospital, Ghent	Allogenic vs Autologous FMT	yes	Phase 2/3	Any MDRO	150	NCT04188743		Unknown	
2019	University Health Network, Toronto	FMT	yes	Phase 2/3	CRE	40	NCT03802461		Ongoing	
2019	Rambam Health Care Campus	FMT	no	N/A	CRE	60	NCT03391674		Ongoing	
2019	University of British Columbia	FMT	no	N/A	Any MDRO	90	NCT04181112		Ongoing	
2019	Vancouver Island Health Authority	FMT	no	N/A	Any MDRO	50	NCT03834051		Ongoing	
2019	Raymond Poincaré Teaching Hospital	FMT (nasoduodenal tube)	no	N/A	VRE	8		++++	87% decolonization after 3 months	[30]
2019	Saint Antoine Hospital, Paris	FMT (enema or nasogastric tube)	no	N/A	CRE/VRE	10		+++	Generally safe and effective in these patients	[25]
2019	University of British Columbia	FMT (enema) +/− antibiotic	no	N/A	MDROs	90	NCT04181112		Ongoing	
2019	Geneva University Hospitals	FMT (pill and nasopharengeal)	yes	Phase 2	ESBL/CPE	39	NCT02472600	+	Slight reduction in colonization	[23]
2020	Rambam Health Care Campus	FMT (pills)	yes	Phase 2/3	CRE	60	NCT04146337		Not yet recruiting	
2022 *	University of Wisconsin, Madison	FMT (pills)	yes	Phase 2	CRE/VRE	90	NCT03643887		Not started	

Fecal microbiota transplantation (FMT), *Lactobacillus rhamnosus* GG (LGG), vancomycin-resistant enterococci (VRE), methicillin-resistant *Staphylococcus aureus* (MRSA), multidrug resistant (MDR), *Lactobacillus casei rhamnosus* (Lcr35), carbapenem resistant *Klebsiella Pneumonia* (CRKP), extended spectrum beta-lactamases (ESBL), carbapenem-resistant enterobacteriaceae (CRE), extended-spectrum resistant (ESC-R), urinary tract infections (UTIs). <25% improvement (+), 26% to 50% improvement (++), 51% to 75% improvement (+++), 76% to 100% improvement (++++). * Projected start date.

**Table 2:** Companies developing microbiome-modifying therapies that could be used for MDROs.

Company	Product Name or Prefix	Therapy Type	Proposed Mechanism	Trials Specifically for MDRO
Rebiotix	RBX2660	FMT (enema)	Displacement of MDRO	Phase 1/2
Kaleido	KB109	prebiotic	Feed healthy bacteria to out-compete MDRO	Clinical Food Study
ExeGi	Visbiome (US), Vivomixx (EU)	probiotic	Displacement of MDRO	Yes [31]
Vedanta	VE707	rationally selected microbiota	Displacement of MDRO	Preclinical
SciBac	SCB	engineered probiotic	Transfer of plasmids to enhance good bacteria	Preclinical
Rise Therapeutics	R	delivery technology for protein therapies	Immune modulation	Preclinical
Finch	CP & FIN	FMT, rationally selected microbiota	Displacement of MDRO	No
OpenBiome	unbranded pills	FMT (pills)	Displacement of MDRO	No
Seres	SER	FMT, rationally selected microbiota	Displacement of MDRO	No
Evelo	EDP	monoclonal microbials	Immune modulation	No
Enterome	EB	small molecule	Immune modulation	No
PureTech Health	numerous	hydrogel	Physical clearing of gut	No
Atterx	C-1205	lyophilized *E. coli*	Prevents growth of MDRO	On website, no NCT
Atterx	GN-4474	bacterial conjugation + killer plasmid	Transfer of toxic plasmid to target bacteria	On website, no NCT

**Table 3:** Clinical trials for probiotics and prebiotics that directly target MDRO colonization or infection.

Treatment Type	Year	Sponsor	Product	Placebo/Controlled	Clinical Stage	Target	Number of Patients	NCT Number	Effect	Conclusions	Reference
Probiotic (live bacteria product)	2000	Karolinska Institutet	probiotic or vancomycin	no	N/A	Healthy volunteers	40		0	No effect on VRE colonization	[40]
	2003	Tufts Medical Center	LGG or Culturelle	yes	Phase 1	VRE	11	NCT00756262	0	No effect on VRE colonization	[43]
	2003	University Hospital, Clermont-Ferrand	Lcr35	unclear	N/A	MDR *Pseudomonas aeruginosa*	400	NCT00621803		Terminated	
	2007	Austin Health	yogurt with LGG	yes	N/A	VRE	27		++++	100% cleared	[49]
	2007	Oregon Health and Science University	yogurt drink	yes	N/A	VRE	8	NCT00591474		Terminated	
	2008	University of Wisconsin, Madison	VSL#3	yes	Phase 2	VRE	50	NCT00933556	0	No effect on VRE but tolerated probiotics well	[42]
	2008	Hadassah Medical Organization	VSL#3	yes	Phase 1/2	CRKP	60	NCT00722410		Unknown	
	2009	University Hospital, Clermont-Ferrand	Lcr35	yes	Phase 4	VRE	24	NCT00437580		Unknown	
	2009	Bio-K Plus International	*L. acidophilus* CL1285®	yes	Phase 2	MRSA	146	NCT00941356		Unknown	
	2010	University of Wisconsin, Madison	*L. rhamnosus* HN001	yes	Phase 2	MRSA	49	NCT01112995	+	30-50% reduction at 4 weeks	[41]
	2011	University of Otago	*E. coli* Nissle 1917 (Mutaflor)	yes	N/A	MDR *E. coli*	69		0	No effect	[44]
	2011	Baskent University	*L. reuteri*	yes	N/A	Any MDRO	76	NCT02178267	0	No difference in acquisition of MDROs	[47]
	2011	Poznan University of Medical Sciences	LGG	yes	N/A	VRE (children)	61		+/-	Temporary elimination of VRE	[51]
	2011	University of Wisconsin, Madison	*L. rhamnosus* HN001	yes	Phase 2	MRSA	113	NCT01321606	+	Reduced odds of MRSA colonization of the gut	[50]
	2012	Washington University School of Medicine/ CDC	Culturelle (LGG)	yes	Phase 4	Any MDRO	103	NCT01551186	0	No effect of acquisition or loss of any MDROs	[46]
	2014	Washington University School of Medicine/ CDC	Culturelle (LGG)	yes	Phase 4	Any MDRO	87	NCT02046512	0	No effect of acquisition or loss of any MDROs	[46]
	2014	Hospital Universitario La Paz	Lactitol and Lactobacillus	no	Phase 2	CRKP	20	NCT02307383		Suspended (Unable to recruit patients)	
	2016	Universidade de São Paulo	symbiotic product	yes	N/A	MDR Gram-negative	101		0	Not effective for decolonizing	[45]
	2017	Lund University/ ExeGi	Vivomixx (EU) /Visbiome (US)	yes	N/A	ESBL	80	NCT03860415	0	Vivomixx® was not superior to placebo	[31]
	2018	Procter and Gamble/ Ochsner Health System	*B. infantis*	yes	N/A	MDR urinary tract infections	100	NCT03644966		Ongoing	
	2018	Hvidovre University Hospital	Lactobacillus	yes	N/A	VRE	162	NCT03560700		Not yet recruiting	
	2019	Aarhus University Hospital	LGG	yes	N/A	MDR Enterobacteriaceae	61		0	No difference in colonization	[48]
	2019	Hospital Italiano de Buenos Aires	probiotic	yes	N/A	CRE	228	NCT03967301		Ongoing	
	2019	Taipei Medical University /Delta Electronics	probiotic cocktail	yes	N/A	VRE	100	NCT03822819		Ongoing	
	2020	University of Bergen	Labinic (R) probiotic	yes	Phase 3	ESBL	2000	NCT04172012		Ongoing	
Prebiotic/food	2019	Columbia University	Inulin	yes	Phase 2	Any MDRO	90	NCT03865706		Ongoing	
	2019	Kaleido Biosciences	KB109	yes	N/A	VRE, ESBL, or CRE	64	NCT03944369		Ongoing	

Fecal Microbiota Transplant (FMT), *Lactobacillus rhamnosus* GG (LGG), vancomycin-resistant enterococci (VRE), methicillin-resistant *S. aureus* (MRSA), multidrug resistant (MDR), carbapenem-resistant enterobacteriaceae (CRE), extended spectrum beta-lactamases (ESBL), *Lactobacillus casei rhamnosus* (Lcr35), and carbapenem resistant *Klebsiella Pneumonia* (CRKP). No effect (0), mixed results (+/−), <25% improvement (+), 76% to 100% improvement (++++)
